# The size of the attentional window when measured by the pupillary response to light

**DOI:** 10.1038/s41598-018-30343-7

**Published:** 2018-08-08

**Authors:** Shira Tkacz-Domb, Yaffa Yeshurun

**Affiliations:** Psychology Department and Institute of Information Processing and Decision Making, University of Haifa, Haifa, 3498838 Israel

## Abstract

This study measured the size of the attentional window when attention is narrowly focused, using attentional modulation of the pupillary light response – pupillary constriction when *covertly* attending a brighter than darker area. This allowed us to avoid confounds and biases involved in relying on observers’ response (e.g., RT), which contaminated previous measurements of this window. We presented letters to the right and left of fixation, each surrounded by task-irrelevant disks with varying distances. The disks were bright on one side and dark on the other. A central cue indicated which letter to attend. Luminance levels were identical across trials. We found that pupil size was modulated by the disks’ luminance when they were 1° away from the attended letter, but not when this distance was larger. This suggests that the diameter of the attentional window is at least 2°, which is twice as large as that established with behavioral measurements.

## Introduction

Spatial covert attention refers to processes that prioritize information gathered from a specific location, in the absence of eye movements. The attentional window is defined as a restricted region in our visual field to which spatial attention is deployed. Many studies examined the characteristics of the attentional window. For instance, several studies suggested that the size of the attentional window varies with task demands^1^. Others showed that the size of the attentional window can be voluntarily modulated^2,3^. Additionally, it was suggested that the size of the attentional window plays a critical role in attentional capture, as attention can only be captured by stimuli appearing within the attentional window^4,5^. Eriksen and Hoffman^6^ further suggested that the size of the attentional window can be narrowed down to a diameter of about 1° of visual angle (i.e., 0.5° from center to edge). Critically, most of the previous studies explored the characteristics of the attentional window using behavioral measurements, particularly reaction time (RT). For example, Eriksen and Hoffman^6^ presented a target letter flanked on each of its sides by two irrelevant letters (distractors). Target and distractors were chosen from the same set of four letters (A, H, U, and M), and target identification RT was measured under three target-distractor distances: 0.53°, 1.0°, and 1.4°. They found no significant difference between the second and third distance conditions, but RT was significantly slower with the smallest distance. This led Eriksen and Hoffman to conclude that only with the distance of 0.53° the distractors were included within the attentional window. However, behavioral measurements could be affected by various factors that are not directly related to the size of the attentional window, such as higher-level strategies, decisional criteria, experience, learning, speed accuracy tradeoffs, response biases, etc. This is particularly so with RT, as it also involves factors related to motor preparation. The goal of the current study, therefore, was to estimate the minimal size of the attentional window (i.e., the size of the attentional window when attention is narrowly focused on the target), with a measurement that is independent of performance – the pupillary light response (PLR).

It is well known that the pupils change their size in response to light – they constrict when light levels are high and dilate when light levels are low^7^. For many years the PLR was considered a reflex that is mediated by low level, subcortical, mechanisms^8,9^. Interestingly, recent studies demonstrated that spatial covert attention can also affect the PLR. Under identical overall luminance conditions, covertly shifting spatial attention to a brighter area produces pupillary constriction relative to shifting covert attention to a darker area^10–14^. For instance, Binda *et al*.^10^ presented two disks, each with a dot at its center, against a uniform gray background. One disk was presented to the right and the other to the left of a central fixation mark. Additionally, one disk was bright and the other one was dark. A central cue instructed observers to covertly attend one of the disks while fixating the central fixation mark. The task was to count the number of times the dot on the attended disk changed its color. The results showed that pupil size depended on where spatial covert attention was focused: when the bright disk was attended pupil size was smaller relative to when the dark disk was attended. Similar results were found by Mathôt *et al*.^11^ and further extended to involuntary allocation of covert spatial attention^12^. We used these attentional modulations of the PLR to assess the spatial extent of attention without having to rely on performance. Specifically, we employed a task requiring the participants to focus their attention narrowly on a target, and added to the display task-irrelevant stimuli of different levels of luminance. The irrelevant stimuli were presented at varying distances from the target. As long as these distances were small enough so that the irrelevant stimuli fell within the attentional window, we expected to find a brightness effect: smaller pupil size when covert attention is directed to a target surrounded by bright than dark irrelevant stimuli. Once the irrelevant stimuli appear at a distance larger than the attentional window, their luminance should not affect pupil size. This allowed us to estimate the size of the attentional window.

**Figure 1:**
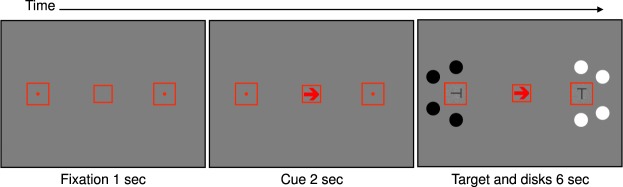
Stimuli and sequence of events for a single trial in Experiment 1. Each trial started with an isoluminant red fixation mark and two peripheral squares. An arrow (the cue) followed, instructing observers which side to attend. Next, the Ts and disks appeared simultaneously and the Ts rotated for 6 sec. The distances of the disks from each T were varied to derive the attentional window size. Each distance was kept constant during a block of 56 trials and varied randomly across different blocks. Cue direction, the side at which the bright and dark disks appeared, and target-disks distances varied randomly within a block.

We conducted two such experiments, in which we presented two squares to the right and left of the fixation (Fig. 1). Each square included a rotating T. Task-irrelevant disks surrounded each square with varying inter-stimuli distances. The disks were bright on one side and dark on the other, thus the overall luminance of the screen was identical on all trials. On each trial a central cue instructed observers to covertly shift attention to the right or left square. The target was the T on the attended side. The Ts on both sides changed their orientation randomly and independently. The task was to count the number of times the attended T assumed an upright orientation, without moving the eyes from the center of the screen. Therefore, this task imposed a narrow attentional focus.

## Experiment 1

### Methods

#### Observers

Twelve observers participated in this experiment. All observers were students from the University of Haifa, with normal vision and without diagnosis of attention deficit disorder. All observers signed an informed consent form. Observers were naive to the purpose of the study and were either paid or received course credit for participating. This study adhered to the Declaration of Helsinki, and was approved by the ethics committee of the University of Haifa.

#### Stimuli and apparatus

Stimuli were presented using the Psychophysics Toolbox extensions^15^ in MATLAB on a 19-in. monitor of an IBM-compatible PC (1,024 × 768 resolution at refresh rate of 85 Hz). Pupil diameter and eye movements were recorded from the right eye with an EyeLink 1000 eye tracker (temporal resolution of 1000 Hz; SR Research, Ottawa, ON, Canada). Two squares (2° × 2°, each) were presented at an eccentricity of 6.75° to the right and left of a central square fixation mark (1° × 1°). An arrow (0.7° × 0.7°) pointing to the right or the left appeared inside the central square, and a rotating letter T (1° × 1°) was displayed inside each of the other squares against a static random dot noise that filled each square (dark dots: 11.5 cd/m^2^; bright dots: 37.5 cd/m^2^). The Ts could be oriented upright, inverted, 90° to the right or to the left, and they changed their orientation randomly and independently. The luminance of the Ts was set individually for each observer to avoid performance that is too high or too low (luminance range: 15–19 cd/m^2^; luminance mode: 19 cd/m^2^). This was established based on the practice session. If performance was either very high or very low in the practice session the experimenter changed the luminance of the T in the experimental session accordingly to make the task harder or easier (increased or decreased, respectively). The contours of all squares and the arrow were colored in isoluminant red (24.5 cd/m^2^), and the background was a uniform gray (24.5 cd/m^2^). A group of four disks surrounded each T with varying inter-stimuli distances. The distances from the center of each T to the inner edge of the disks that appeared above and below the T were 1°, 4°, 7° and 11° of visual angle. The distances of the disks that appeared diagonally above and below each T were 1.5°, 4.5°, 7.5° and 11.5°. Each disk had a diameter of 1°. The disks were bright (90 cd/m^2^) on one side of the screen and dark (0.04 cd/m^2^) on the other side.

#### Procedure

At the beginning of the session, observers were instructed to look only at the center and avoid blinking throughout each trial, but were encouraged to take short breaks between trials and long breaks between blocks. Then, a 9 points calibration was conducted. Each trial was preceded by a drift check to ensure the accuracy of the calibration is maintained and that the participant looks at the center. A trial started with the central fixation mark and the squares. After 1 sec, the arrow appeared inside the central square indicating the side to which the observers have to attend, and it was presented till the end of the trial. After 2 sec, the disks and rotating Ts appeared simultaneously. The Ts changed their orientation every 500 ms, and were visible for 6 sec. The target was the letter T on the attended side, and the task was to count the number of times the attended target assumed an upright orientation, while looking at the center of the fixation mark. Following a response, an auditory feedback indicated whether or not the response was correct. The distance between the target and disks was constant within a block and varied between blocks. The experiment included 448 experimental trials. For 10 out of the 12 participants these 448 experimental trials were divided into 4 sessions of 112 trials. These sessions were conducted on different days to minimize fatigue or at the same day if the participant could rest for at least two hours between sessions. In each session there were two blocks, each of a different target-disks distance. The order of distances was random, but to avoid order effects each distance appeared once as the first block of a session and once as the second block of a session. For 2 observers the 448 experimental trials were divided into 2 sessions of 224 trials, which included 4 blocks each. For all 12 participants, each experimental session was preceded by a practice session that included 20 practice trials. These practice trials did not include the disks in order to motivate the observers to concentrate only on the task. In addition, before the beginning of each experimental block, there were 5 practice trials that were identical to those of the upcoming experimental block. The side at which the bright (dark) disks were displayed, and the side to which the arrow pointed were chosen randomly for each trial and were counterbalanced across the entire experiment.

### Results

#### Data analysis

We included in the analyses only trials in which fixation was not broken more than 1° and pupil size ranged between 2 to 8 mm^10^ (average percentage of trials excluded: 11.1%). At first, blinks events were linearly interpolated^16,17^. Then, the sampling points of pupil size were averaged within 50 ms time bins. The baseline was the average pupil size of the 100 ms preceding the onset of the disks and Ts. For each trial, the average pupil size of each bin was divided by the baseline value. We performed a linear mixed effects analyses (LME) for each bin, using R’s *lme4* package^18,19^. The variable of pupil size was entered into the model as the dependent measure and the variable of subjects served as a random effect. To test for a difference between the disk brightness conditions (i.e., attended target surrounded by bright disks vs. attended target surrounded by dark disks; which we will refer to as ‘brightness’), as well as the effect of target-disks distance, the variables of brightness and target-disks distance as well as their interaction were entered to the model as fixed effects. Additionally, to examine in more details the relationship among these two variables, we conducted a separate analysis for each target-disks distance with disks brightness at attended side as a fixed effect. Similar to previous studies, we defined the significance criterion as *t* > 2 for at least 200 ms time interval^11,12,20,21^.

#### Data availability

The data that support the findings of this study are available in the Open Science Framework repository, https://osf.io/tg5n9/?view_only=3039239e39104ee292700b5cdf022f8c.

**Figure 2:**
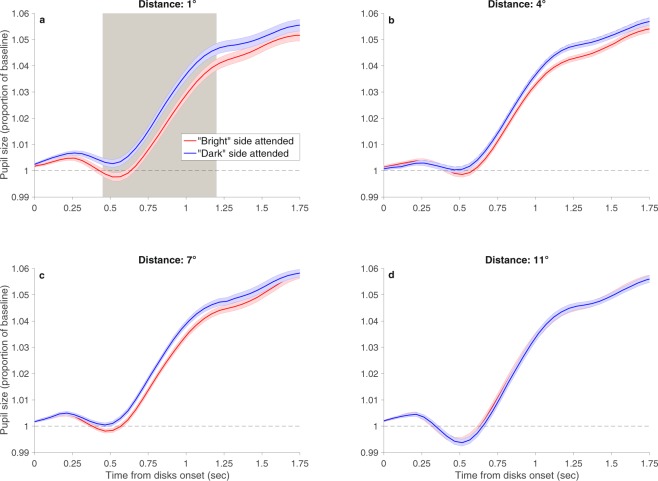
Results of Experiment 1. Pupil size (calculated as proportion of baseline) as a function of time from disks onset. The baseline was the average pupil size over the last 100 ms before disks onset. (**a**) Target-disks distance of 1°; (**b**) target-disks distance of 4°; (**c**) target-disks distance of 7° and (**d**) target-disks distance of 11°. With the distance of 1°, the effect of disks brightness at the attended side was significant from 0.45 to 1.2 sec, as indicated by the gray shading. The interaction was significant from 0.55 to 0.95 sec. Colored shadings indicate one within-subject standard error^45^. For the sake of clarity, the figure depicts only the time interval of the trial in which significant effects were found.

The results are presented in Fig. 2. For all four distances, the onset of the target and disks evoked a transient constriction of the pupil, regardless of disks brightness. This is consistent with previous studies^22,23^. In addition, with all four distances there was a gradual increase in pupil size as the trial advanced, regardless of disks brightness. Such gradual increase was also demonstrated before, and it is probably due to cognitive effort associated with the task^24,25^. More important for the goal of this study, we found a significant interaction between brightness and distance. Further analyses performed for each distance separately revealed a significant brightness effect with the smallest distance of 1°: Pupil size was smaller when the attended target was surrounded by bright than dark disks. Critically, this difference was not significant at any time along the trial with the other larger distances. Hence, the results suggest that disks that were presented at a distance of 1° from the target fell within the attentional window, whereas disks that were presented at a distance of 4° or larger fell outside the attentional window.

## Experiment 2

To establish a more accurate estimation of the attentional window, we performed Experiment 2, which was similar to Experiment 1, but examined the attentional effect on the PLR with a finer scale of distances (1°, 2°, 3° and 4°).

### Methods

#### Observers

Twelve observers participated in this experiment, of which 6 also participated in Experiment 1. All observers were students from the University of Haifa, with normal vision and without diagnosis of attention deficit disorder. All observers signed an informed consent form. Observers were naive to the purpose of the study and were either paid or received course credit for participating. This study adhered to the Declaration of Helsinki, and was approved by the ethics committee of the University of Haifa.

#### Stimuli, apparatus and procedure

The stimuli, apparatus and procedure were similar to Experiment 1 except for the following. The luminance of the Ts ranged between 15 to 21 cd/m^2^ in Experiment 2 with a mode of 19 cd/m^2^. The distances from the center of each T to the inner edge of the disks that appeared above and below the T were 1°, 2°, 3° and 4°, and from the diagonally upper and lower disks were 1.5°, 2.5°, 3.5° and 4.5°. The presentation duration of the Ts was shortened to 4 sec. This was motivated by the fact that no effects of interest emerged at the last 2 sec of the trials in Experiment 1. With all the participants, the 448 experimental trials were divided into 4 sessions of 112 trials, as described for Experiment 1.

### Results

**Figure 3:**
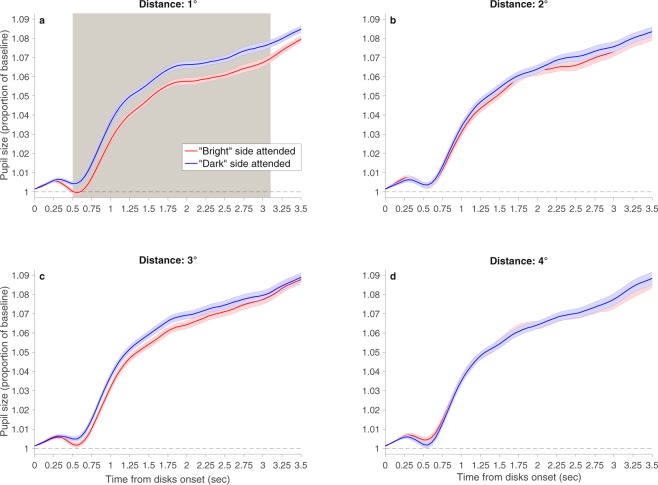
Results of Experiment 2. Pupil size (calculated as proportion of baseline) as a function of time from disks onset. The baseline was the average pupil size over the last 100 ms before disks onset. (**a**) Target-disks distance of 1°; (**b**) target-disks distance of 2°; (**c**) target-disks distance of 3°; and (**d**) target-disks distance of 4°. The gray shading indicates the times in which the disks brightness effect was significant. With the distance of 1°, this effect was significant from 0.5 to 3.1 sec. The interaction was significant from 0.55 to 1.2 sec. Colored shadings indicate one within-subject standard error^45^. For the sake of clarity, the figure depicts only the time interval of the trial in which significant effects were found.

Statistical analyses were identical to those of Experiment 1 (average percentage of trials excluded 7.8%). A similar significant brightness × distance interaction emerged (Fig. 3): With the smallest distance of 1°, pupil size was significantly smaller when the attended T was surrounded by bright than dark disks. In contrast, with the other distances, this difference was not significant at any time throughout the trial.

## Discussion

The results of both experiments demonstrate that disks that where presented at a distance of 1° from the target fell within the attentional window, even though they were task-irrelevant. If the attentional window is viewed as a round window centered at the target, then our findings propose that the diameter of this window is at least 2°. This suggests that the minimal size of the attentional window is twice as large as that found with behavioral measurements^6^. Experiment 2 further shows that disks that where presented at a distance of 2° or more, fell outside the attentional window, suggesting that the diameter of the attentional window is smaller than 4°. It is important to note that the distribution of attention is likely not uniform but rather falls gradually from the center of the attentional focus^26,27^, and that there is evidence for individual differences in the spreading of attention^28,29^. Such individual differences and general noise in the data may deem impractical an attempt to establish a finer estimation of the size of the attentional window, at least when looking for the minimal size.

A substantial part of our knowledge about spatial covert attention was established in studies that inferred attention allocation based on differences in performance. This is especially so regarding experiments that attempted to measure the size of the attentional window^1,6,30–33^. The typical way by which these studies measured the size of the attentional window was to measure distractor interference as a function of target-distractor distance; when distracting stimuli influenced performance it was concluded that they fell inside the attentional window^6,32,33^. However, this conclusion was debatable, because a large body of evidence suggests that unattended visual stimuli are also processed and can affect performance^34–37^. Hence, the mere fact that performance was affected by distracting stimuli does not necessarily mean that these stimuli fell within the attentional window. Likewise, the lack of response interference may not necessarily mean that the distracting information fell outside the attentional window. For example, Merikle and Gorewich^38^ suggested an alternative critical factor –the size of the distractors, or more specifically whether or not they meet the limitations of visual acuity. They presented a color name above and below a central color patch with varying distances: 0.5° or 2.5°. The color names were either incompatible with respect to the color patch (e.g., the word “blue” presented with a red color patch) or neutral (a string of the letter ‘l’: “1111”), and they could be small (0.24°) or large (0.57°). Participants had to rapidly identify the color patch while ignoring the color names. The results revealed that when the target-distractors distance was 0.5°, distractor interference emerged for both large and small distractors: RT was significantly slower in the incompatible than neutral condition. However, when the target-distractors distance was 2.5° an interference effect was found only with the large distractors. These findings suggest that the lack of interference effect with the small distractors at the large distance was not necessarily due to the fact that they were presented outside the attentional window. More likely, the lack of effect was due to the fact that at this distance from the center the color names were too small to be resolved, and therefore too small to generate response interference. Likewise, Gatti and Egeth^39^ employed a similar paradigm and found that the interference generated by incompatible color names decreased as the distance between the color patch and color names increased. This led them to conclude that visual acuity plays an important role in distractor interference, and may compromise measurement of the size of the attentional window. Visual acuity limitations are not a problem in our study because we employed relatively large distractors (each disk was 1° in diameter, which is almost twice the size of Merikle and Gorewich’s^38^ large distractors) and there were four such distractors around the target. Moreover, because our method for measuring the size of the attentional window does not rely on response interference generated by distractors with conflicting semantic meaning, our measurements are not affected by the ability/inability to resolve the distractors’ fine details.

As mentioned above, even when distractor interference is found, this by itself does not necessarily mean that the distractors were attended, because unattended stimuli are also processed. Indeed, after conducting an impressive serious of experiments that look at different factors that may affect the processing of the distractors, including their distance from the target, Miller^35^ concluded that: “…complete elimination of unattended stimuli from semantic processing is a rare occurrence, probably requiring a very special combination of circumstances”. (p. 286). Moreover, Driver and Tipper^36^ showed that both distractors that generated response interference and those that did not, produced equivalent negative priming, indicating that both interfering and noninterfering distractors were processed and represented. This finding suggests that the presence or absence of distractor interference cannot serve as an indication of attention allocation or processing level (see Fox^37^ for similar conclusion). The current study overcomes this problem by not relying on differences in performance but on modulations of pupil size. In our study, the disks were neither compatible nor incompatible with the required response, and they were always bright on one side and dark on the other side, so the overall luminance level was always the same and it always affected the PLR. Critically, we tested effects on PLR beyond the overall luminance level – effects that are specific to covert attentional modulations^13^. If the disks were displayed close enough to the focus of attention so that they fell inside the attentional window (i.e., they were part of the attended region) then pupil size was modulated by their brightness. Therefore, the measurement of the PLR seems more informative for our current goal than differences in performance. Additionally, contrary to performance, pupil size does not appear to be under a direct volitional control^7^ and therefore could serve as an uncontaminated measurement of attentional effects.

Prior research of the attentional window suggests that its size, at a given moment, depends on several different factors. For instance, Caparos and Linnell^26^ demonstrated that the characteristics of the attentional window vary as a function of perceptual load; LaBerge^1^ demonstrated that the size of the attentional window varies as a function of task demands; and a study by Sagi and Julesz^31^ showed that the size of the window changes as a function of eccentricity. Previous studies who examined the effects of such factors on the size of the attentional window employed only behavioral measurements. Given the limitations of the behavioral measurements detailed above, our understanding of the characteristics of the attentional window would greatly benefit from future studies that will employ the PLR to carefully examine the various factors that mediate the size of the attentional window.

Taken together, this study has important methodological, theoretical and applied implications. In terms of research methods, this study demonstrates the effectiveness of PLR examination as a method to study the nature of spatial attention – a method that is not contaminated by all the above-mentioned factors that are not directly related to attention. Moreover, the results of this study are important for cases in which it is essential to know the exact size of the attentional window in order to ensure that stimuli are falling/not falling within the attentional window. This is critical for the design of experiments that wish to control which information is attended/unattended at any given moment. Additionally, from a more applied point of view, it is also important for the design of optimal visual displays of any form (e.g., graphical user interface, printed material, vehicles dashboard, etc.), who wish to minimize clutter. Furthermore, our study has important theoretical implications for the development of physiological and computational models of spatial attention, because the minimal size of the attentional window poses a fundamental constrain on such models. For instance, neurophysiological models of attention have to ensure that the neural substrate suggested to underlie spatial attention can support the minimal size of the attentional window. Similarly, in several computational models, such as the normalization model of attention^40^ and the attentional attraction field (AAF) model^41^, a parameter that represents the attentional spread plays an important role. Such models have to ensure that the value they assign to this parameter, when attempting to simulate performance, comply with the minimal size found here. Finally, our findings are important for cross-validation of neurophysiological studies that attempt to examine the characteristics of the attentional window using functional neuroimaging data^42–44^.

To sum, using the attentional modulation of the PLR we found that the minimal size of the attentional window when attention is narrowly focused has a diameter of about 2°, which is twice the size found when measured based on performance differences.
